# Distinct roles of CD4^+ ^T cell subpopulations in retroviral immunity: lessons from the Friend virus mouse model

**DOI:** 10.1186/1742-4690-8-76

**Published:** 2011-09-26

**Authors:** Savita Nair, Wibke Bayer, Mickaël JY Ploquin, George Kassiotis, Kim J Hasenkrug, Ulf Dittmer

**Affiliations:** 1 Institute for Virology, University Clinics Essen, University of Duisburg-Essen, Hufelandstrasse 55, 45122 Essen, Germany; 2 Laboratory of Persistent Viral Diseases, Rocky Mountain Laboratories, NIAID, NIH, Hamilton, MT 59840, USA; 3 Division of Immunoregulation, MRC National Institute for Medical Research, The Ridgeway, London NW7 1AA, UK; 4 Immune Cell Development and Host Defense Program, Fox Chase Cancer Center, 333 Cottman Avenue, Philadelphia, PA 19111, USA

## Abstract

It is well established that CD4^+ ^T cells play an important role in immunity to infections with retroviruses such as HIV. However, in recent years CD4^+ ^T cells have been subdivided into several distinct populations that are differentially regulated and perform widely varying functions. Thus, it is important to delineate the separate roles of these subsets, which range from direct antiviral activities to potent immunosuppression. In this review, we discuss contributions from the major CD4^+ ^T cell subpopulations to retroviral immunity. Fundamental concepts obtained from studies on numerous viral infections are presented along with a more detailed analysis of studies on murine Friend virus. The relevance of these studies to HIV immunology and immunotherapy is reviewed.

## Introduction

CD4^+ ^T lymphocytes are a specialized subpopulation of T cells that recognize antigenic peptides in the context of MHC class II molecules. Historically, CD4^+ ^T cells have been regarded as 'helper' T (Th) cells, since CD4^+ ^T-cell help is required for both the induction of neutralizing antibodies by mature B cells and for the maintenance of effective cytotoxic T cell (CTL) responses. In the mid-1980s functional attributes were discovered that allowed CD4^+ ^T cells to be subdivided into dichotomous subpopulations of Th1 and Th2 cells [1].

Th1 cells are defined by their property to produce IFNγ, TNFα and IL-2 cytokines, and play critical roles in anti-tumor immunity [2] and immune responses to many virus infections including lymphocytic choriomeningitis virus (LCMV) [3], influenza virus [4], vesicular stomatitis virus (VSV) [5], polio virus [6], and murine γ herpes virus [7]. Besides helper functions, Th1 cells also have important effector functions. For example, in addition to their immunoregulatory activities, both IFNγ and TNFα cytokines mediate direct anti-viral activities as observed in murine infections of LCMV [8], herpes simplex virus (HSV) [9], vaccinia virus [10], measles virus (MV) [11] and Friend virus (FV) [12]. Th1 cells may also have cytotoxic potential as observed in a number of viral infections, including dengue virus [13], HSV [14], hepatitis B virus (HBV) [15], MV [16], human herpesvirus 6 [17], HIV [18] and Epstein-Barr virus (EBV) [19].

By contrast, Th2 cells secrete IL-4, IL-5, IL-9, IL-13 and IL-25 when activated in response to bacterial, helminth or parasitic pathogens such as *Clostridium tetani*, *Staphylococcus aureus*, *Streptococcus pneumonia*, *Pneumocystis carinii, Schistosoma mansoni*, and *Trichinella spiralis *[20]. Th2 cells provide help for B cells to produce IgM, IgA, IgE, and IgG isotype antibodies, which form the effector molecules of the humoral immune response [21].

The Th1/Th2 paradigm introduced by Mossman and Coffman has been expanded by identification of other CD4^+ ^T cell sub-populations. IL-17 secreting cells designated as Th17 cells [22,23] are important for resistance to extracellular bacteria and fungi, but may also contribute to allergic responses [24] and autoimmune pathogenesis in diseases such as multiple sclerosis, rheumatoid arthritis, psoriasis and inflammatory bowel disease [25]. Yet another sub-population of CD4^+ ^T cells is the follicular helper T (Tfh) cell. Upon antigenic stimulation, Tfh produce IL-21 and home to B cell follicles where they are essential for the differentiation of B cells into germinal center B cells and antibody secreting plasma cells [26,27].

Finally, there is a unique subset of CD4^+ ^T cells called regulatory T cell (Tregs) subset that negatively regulates the immune system and serves to prevent autoimmunity and immunopathology [28]. During many different types of infection natural and/or induced Tregs expand to control the pathogen-specific effector T cell response. Evidence indicates that this negative control mechanism is important in limiting T-cell-mediated collateral damage that may occur during immune responses against microbial pathogens. Along these lines, Tregs inhibit the development of immunopathogenesis in Hepatitis C virus (HCV) infections [29], HSV infections [30,31], and FV infections [32]. On the other hand, Treg-mediated suppression of immune responses may delay pathogen clearance as observed in chronic HCV [33-35], HIV [36], EBV [37], HSV [38], and FV [39] infections. In the same context, Tregs also inhibit anti-tumor immune responses and restoration of anti-tumor immunity requires attenuation of Treg functions [40].

The general importance of CD4^+ ^T cells in human health and immunity was dramatically displayed early in the AIDS epidemic as patients presenting with reduced CD4^+ ^T cell counts developed opportunistic infections. CD4^+ ^T cells, the main targets for HIV infection, are rapidly depleted during HIV infection [41,42], eventually leading to the acquired immunodeficiency syndrome known as AIDS. Loss of antiviral IFNγ production by CD4^+ ^T cells, as well as loss of direct cytotoxic activity against infected cells [43-45], contribute to immunodeficiency, but more important may be the loss of CD4^+ ^T cell helper activity. CD4^+ ^T cell help is necessary for long-term CD8^+ ^T cell memory and the development of high-avidity antibody responses, both of which are deficient in HIV infections [46-48]. Another major factor contributing to HIV-induced immunodeficiency is immune system hyperactivation, which appears to be the result of HIV-induced pathology in the gut-associated lymphoid tissue [49,50]. Damage to the gastrointestinal tract early in HIV infection allows immunostimulatory microbial products such as lipopolysaccharide to translocate into the bloodstream [51]. The resulting non-specific activation of immune cells can cause activation-induced cell death and contribute to HIV-associated CD4^+ ^T cell depletion. This dysregulation of the immune response not only reduces the ability to mount pathogen-specific responses, but can cause immunopathogenic effects. Dysregulation is further exacerbated by the loss of CD4^+ ^Tregs, which would normally dampen immunopathogenic responses [52,53].

The reported loss of CD4^+ ^Tregs from the peripheral blood in HIV patients [54], is associated with an accumulation of these same cells in infected lymphatic tissues, suggesting that Tregs either redistribute to infected tissues, proliferate there, or both [36,55]. Tregs at the sites of infection are associated with dysfunctional CD8^+ ^T cells and can inhibit both HIV-specific CD4^+ ^and CD8^+ ^T cell responses *in vitro *[36,54,56]. Interestingly, HIV-infected patients who exert control over virus loads have lower Treg responses [52], suggesting that Tregs indeed contribute to effector T cell dysfunction and inability to clear the infection.

Acute HIV-1 infection is usually characterized by mild flu-like symptoms and hence, only few patients are diagnosed with acute HIV infection. Thus, there is limited opportunity to study the early immunological responses to HIV infection. Another limitation in HIV research is the lack of a tractable small animal model susceptible to HIV infection. The most widely used model is the infection of macaques with simian immunodeficiency virus (SIV), which is closely related to HIV, and an enormous amount of knowledge has been gained from studies in this model. However, there are limitations in the studies that can be done in non-human primates as compared to a mouse model. For example, there are no colonies of congenic, transgenic, or targeted gene knockout macaques available for study. Since there is no perfect solution for scientists to study HIV infections, the approach has been to gain information from studies in humans, non-human primates, and also mouse models, which are useful for elucidating fundamental concepts in retroviral immunology that may have relevance to HIV infections in humans.

A mouse virus that has been particularly informative is the Friend retrovirus, which has provided information regarding basic mechanisms of immunological control and escape in both acute and persistent retroviral infections. Studies of mice infected with FV have revealed a complex balance of immune responses induced by at least two subsets of CD4^+ ^T cells with opposing effects. On one hand, CD4^+ ^Tfh and Th1 cells coordinate B cell and CD8^+ ^T cell immune responses, and additionally induce direct anti-viral effects fortifying the immunological control of FV [57-59]. On the other hand, CD4^+ ^Tregs down-regulate the immune responses of CD4^+ ^Th cells [32,58] and CD8^+ ^CTLs [39,60-62] thus, prolonging the recovery from acute FV infection, and allowing the establishment of a chronic infection. The interplay of different subsets of CD4^+ ^T cells in FV infection and the relevance to HIV infection in humans will be discussed in this review.

## Friend retrovirus infection of mice

FV was isolated from leukemic mice by Charlotte Friend [63] and has since been used for identifying genes that control susceptibility to viral infection and virus-induced cancer [64]. FV is a retroviral complex comprising Friend murine leukemia virus (F-MuLV), a replication competent helper virus that is nonpathogenic in adult mice, and spleen focus-forming virus (SFFV), a replication-defective virus responsible for pathogenesis [65]. SFFV cannot produce its own particles; so it spreads by being packaged in F-MuLV-encoded particles produced in cells co-infected by both viruses. FV infection induces lethal erythroleukemia in susceptible strains of mice [65]. Recovery from FV-induced disease partly depends upon genes mapped to the MHC (H-2) region on chromosome 17 of the mouse. Resistance of adult mice against FV-induced disease is determined by the presence of the 'b' alleles at the H-2D and H-2A regions, important for the induction of rapid and strong FV-specific CTL and CD4^+ ^T-cell responses, respectively [66]. Mice that are resistant to FV-induced disease are homozygous for the 'b' allele at the H-2A region and display a higher magnitude of CD4^+ ^T-cell responses than FV-susceptible mice that have none or only one 'b' allele in the H-2A region [58]. However, despite protection from FV-induced leukemia, resistant mice are unable to clear the virus completely and remain persistently infected for life [64,67].

In the recent past it was discovered that mouse-passaged FV stocks also contained lactate dehydrogenase-elevating virus (LDV), an endemic mouse virus. LDV interferes with anti-FV immune responses compromising early recovery from FV infection [68,69]. Subsequently, much of the data generated with virus stocks containing LDV have been repeated with FV/LDV- stocks, and in this review we discuss results from experiments performed with both FV/LDV+ and FV/LDV- virus stocks.

## The role of CD4^+ ^T cells in FV infection and vaccination

### Specificity of CD4^+ ^T cells in FV infection

CD4^+ ^T cells are indispensable for natural immunity against FV since the absence of CD4^+ ^T cells during the acute or chronic phase of FV infection causes loss of control over FV replication in resistant mice [57-59,70]. CD4^+ ^T cells mediate immunity during FV as well as FV/LDV+ co-infection, as comparable results are obtained in CD4-depletion experiments using FV alone or FV/LDV+ infected mice [58,70]. Use of congenic recombinant mice allowed the identification of two CD4^+ ^T cell epitopes of the F-MuLV gp70 Env molecule that stimulate CD4^+ ^T cell responses in FV infected mice. One of the epitopes lies in the N-terminal region of F-MuLV *env*_122-141 _(DEPLTSLTPRCNTAWNRLKL) and is presented in the context of H-2 IA^b ^molecules while the second epitope is in the C-terminal region of F-MuLV *env*_462-479 _(HPPSYVYSQFEKSYRHKR) and is presented in the context of H-2 IE^b/d ^molecules [71-73]. In addition, an A^b/k ^or E^b/k ^restricted CD4^+ ^T cell epitope in the p15 (MA) region of the F-MuLV *gag*_83-97 _(IVTWEAIAVDPPPWV) protein [74] is associated with the induction of effective CD4^+^T cell immune responses against FV challenge.

### Role of CD4^+ ^T cells in vaccine-induced protection against FV

Protection from FV infection can be elicited by several different types of vaccines including killed and attenuated viruses, viral proteins, peptides, and recombinant vaccinia or adenovirus vectors expressing FV genes. Vaccination with recombinant vaccinia viruses using different combinations of FV protein fragments identified protective epitopes in the F-MuLV Gag and Env proteins, although vaccination with F-MuLV Env vectors protects better against infection than vaccination with a gag vector alone [75,76]. These studies were done in congenic mice to eliminate host genes as variables affecting protection. Adenovirus vectors expressing F-MuLV Env and Gag also induce varying degrees of protection against FV, which can be significantly improved by adding vectors that not only expresses F-MuLV proteins but also displayed F-MuLV gp70 on the viral surface [77]. In these experiments, protection correlated with an enhanced neutralizing antibody and FV-specific CD4^+ ^T cell response after virus challenge. Immunization with synthetic peptide vaccines containing the CD4^+ ^T cell epitopes *env*_121-141 _or *env*_462-479 _from the gp70 Env glycoprotein of F-MuLV induces protection in most of the vaccinated mice [78]. Surprisingly, it was suggested that the protective effect of the CD4 epitope vaccine was dependent on NK cells, as NK cell depletion after vaccination abolished the effect of peptide immunization [79].

Studies using congenic and congenic recombinant mice have demonstrated that the MHC background of the mice used for immunization plays an important role in determining the efficacy of vaccines [64,80]. As expected, only mice expressing MHC class II alleles such as H-2A^b^, which can present the immunodominant CD4^+ ^T cell epitopes are protected when immunized with vaccinia virus recombinants expressing F-MuLV Env protein [71,81]. Of note, recovery of immunized mice from challenge with pathogenic FV requires induction of neutralizing antibodies (IgG) and virus-specific T cell responses [75,81]. The requirement for complex immune responses in inducing protection against FV was confirmed using a live attenuated FV vaccine. Nonpathogenic F-MuLV, which replicates poorly in adult mice, was used as attenuated vaccine. Further attenuation of the virus was achieved by crossing the Fv-1 genetic resistance barrier in mice [82]. Adoptive transfer experiments between congenic mice illustrated that the sterilizing immunity induced by this vaccine depends on virus-specific CD4^+ ^and CD8^+ ^T cell as well as on B cell responses [83]. Whereas the CD8^+ ^T cells and antibodies have some protective activity on their own, vaccine-primed CD4^+ ^T cells alone did not induce protection [84], suggesting that their role in protection against FV is mainly to provide help for effector B and T cell responses. However, high numbers of FV-specific CD4^+ ^T cells mediate direct antiviral effects even in the absence of effector CD8^+ ^T or B cells [59].

### Helper functions of CD4^+ ^T cells in FV infection

**Figure 1:**
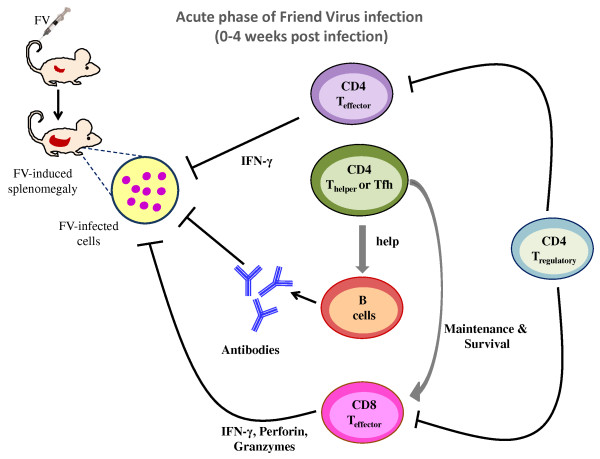
**Distinct populations of CD4^+ ^T cells regulate the virus-specific immune response during acute Friend Retrovirus infection**. CD4^+ ^helper T cells and follicular helper T cells augment virus-specific cytotoxic T cell and antibody responses. In addition, a subpopulation of effector CD4^+ ^T cells directly inhibits virus replication. However, at the same time natural regulatory T cells expand and start to suppress effector T cell responses, which interferes with control of virus replication. (Arrows indicate enhancement of responses, whereas blocked lines indicate inhibition).

Antibodies are critical for most effective antiviral immune responses and utilize a number of different mechanisms to mediate protection. These include blockade of receptor-binding proteins on viruses, lysis of virally infected cells, and lysis of the viruses themselves [85-88]. Passive immunization studies demonstrated that antibodies alone, at concentrations inducible by vaccines, reduce virus loads in FV infected mice but cannot completely prevent infection [83,89]. At these physiological concentrations of antibody, the mice also require T cell-mediated immune responses for protection. The development of effective antibody responses against most viruses, including FV requires help from CD4^+ ^T cells [58,90], and recent evidence indicates that a specialized subset called Tfh cells is essential for B cell help (Figure 1).

The differentiation of Tfh is controlled by expression of the B cell lymphoma 6 (Bcl-6) gene [91-93], and Tfh express several distinct molecules involved in B cell help including CXCR5, PD-1, ICOS, CD40L and OX40. Recent analysis of the differentiation of virus-specific CD4^+ ^T cells during FV infection revealed a prominent Tfh profile [94]. At the peak of the response, up to 40% of the virus-specific CD4^+ ^T cells in the spleen were defined as Tfh by expression of a combination of surface markers (CXCR5, PD-1 and ICOS), transcription factors (Bcl-6) and by their cytokine profile (IL-21). In contrast, little differentiation of virus-specific CD4^+ ^T cells towards Th2, Treg, or Th17 subsets was observed. These studies were made possible with the use of mice carrying a transgenic T cell receptor chain specific for FV. The strong Tfh differentiation of FV-specific CD4^+ ^T cells can be a result of the specific cytokine environment that this infection creates, as it is likely to be the case for Th1 differentiation. Also, the efficient infection of B cells by FV [68,95], which then present FV antigens to specific CD4^+ ^T cells, may contribute to enhance Tfh differentiation [96].

Although Tfh differentiation probably requires high-avidity TCR interactions with antigen-presenting cells following peptide immunization [97], no such requirement is observed during acute FV infection [94]. This finding indicates that levels and/or persistence of antigen presentation during viral infections may exceed those achieved by peptide immunization, and therefore the requirement for high-avidity TCR signaling is bypassed. In HIV infections, the relative control of viremia is associated with the presence of IL-21-producing CD4^+ ^T cells [98]. Interestingly, evidence suggests that IL-21-producing CD4^+ ^T cells may be critical for the maintenance of CD8^+ ^T cell responses during chronic virus infections [99-101], although it remains to be determined whether in all these cases IL-21 is produced by Tfh cells or another T cell subset.

It is known that CD4^+ ^T cells are generally important for the clonal expansion, development of effector function, and the generation of long-term memory CD8^+ ^T cells [102]. The requirement of CD4^+ ^T cell-help for primary CD8^+ ^T cell responses is determined by the nature of the infectious agent and the inflammatory milieu formed by the pathogen [103-105]. Although T cell help may be dispensable in the priming phase of the CD8^+ ^T cell response, it is essential in the generation and maintenance of long-lived memory CD8^+ ^T cells [106-109], and the function of CD8^+ ^T cells during chronic infection [110]. During the first two weeks of acute FV infection the priming and expansion of CD8^+ ^T cells occurs independently of CD4^+ ^T-cell help [68]. In contrast, CD4^+ ^T cells are required for the maintenance of effector and memory FV-specific CD8^+ ^T cells during the recovery phase of FV infection [58] (Figure 1). The situation is slightly different in HIV-1 infections where the development of effector CD8^+ ^T cell responses is compromised in the absence of help from CD4^+ ^T cells [47]. As mentioned above, there appears to be a role for CD4^+ ^T cell-produced IL-21 in the development of HIV-specific CD8^+ ^T cell responses [98], and IL-21 has also been shown to be an important cytokine in the maintenance of CD8^+ ^T cell functionality during chronic viral infections [99-101].

### Direct anti-viral functions of CD4^+ ^T cells against FV

**Figure 2:**
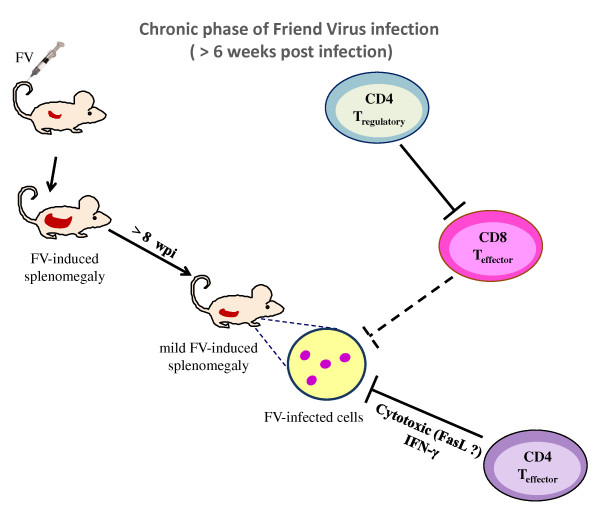
**Distinct roles for CD4^+ ^T cell subpopulations in chronic Friend Retrovirus infection**. During chronic infection, effector CD8^+ ^T cell responses are suppressed by regulatory T cells but a subpopulation of effector CD4^+ ^T cells prevents virus reactivation. (Blocked lines indicate inhibition of immune responses or virus replication; the dotted line indicates that this response is suppressed during chronic infection).

In addition to classical helper functions, CD4^+ ^T cells possess direct effector functions important in controlling infectious agents. As demonstrated *in vitro*, IFNγ secreted by CD4^+ ^Th1 cells during FV infection is a key component involved in the direct anti-viral effects of CD4^+ ^T cells [12]. Studies in genetic knockout mice and mice depleted of IFNγ-producing CD4^+ ^T cells suggest an especially important role in the long-term control of persistent FV infection [12,57,111,112] (Figure 2). FV-specific CD4^+ ^T cells from CD4^+ ^TCRβ-transgenic mice with a TCRβ chain specific for the F-MuLV *env*_122-141 _epitope rapidly expand in an antigen-dependent manner when adoptively transferred into acutely infected mice. The cells differentiate into Th1-type effector CD4^+ ^T cells that produce IFNγ [58,59] (Figure 1). Adoptive transfers of FV-specific CD4^+ ^T cells into FV-infected mice that are either lymphocyte-deficient or depleted, protect from acute disease even in the absence of cytotoxic T cell or antibody responses [59]. These results indicate potent and direct anti-viral effects by CD4^+ ^T cells. Protection is not solely based on IFNγ production, since protection against acute disease is also seen in IFNγ receptor deficient mice [59]. However, FV-specific CD4^+ ^T cells only protect immunodeficient mice against acute disease, and all animals eventually succumb to the infection in the absence of CD8^+ ^T cells and B cells [59]. In HIV infection too, anti-viral effector responses in HIV-1-infected long-term non-progressors are associated with increased levels of IFNγ, the chemokine RANTES, and the macrophage inflammatory proteins MIP-1α and MIP-1β that are produced by virus-specific CD4^+ ^T cells [113]. The rare individuals who display immunological control over HIV not only possess effective CD8^+ ^CTL [114,115], but also contain multiple CD4^+ ^T cell clones with the characteristics of highly efficient effector cells that have high-avidity to HIV *gag *peptides and produce IFNγ [116]. A most interesting and poorly understood aspect of HIV controllers is that they can maintain cell-mediated immune responses over long periods of chronic infection, a situation where most cell-mediated responses become exhausted and ineffective.

In addition to providing help and secreting antiviral factors, it has also been shown that CD4^+ ^T cells can develop the capacity to lyse infected cells. Although most data come from cell lines and CD4^+ ^T cell clones, it has been shown that CD4^+ ^T cells specific for LCMV [117], influenza [118] have cytotoxic activity *in vivo*. Furthermore, cytotoxic CD4^+ ^T cells from the peripheral blood of individuals infected with HIV-1, influenza, EBV or CMV display cytotoxic activity directly *ex vivo *[119-124]. One obvious limitation on CD4^+ ^T cell-mediated cytotoxic activity is that cognate antigen is only recognized on target cells that express MHC class II molecules. Direct antiviral activity by CD4^+ ^T cells seems to be critical during chronic FV infection while the presence of virus-specific CD8^+ ^T cells and virus-neutralizing antibodies have no correlation with chronic virus control (Figure 2). FV replicates mainly in nucleated erythroid precursors which are MHC class II-negative, and cytolysis of these cells is only observed as a by-stander effect in the presence of APCs [12]. However, MHC class II-positive B cells are the main reservoirs of persistent FV [57] and are susceptible to CD4^+ ^T cell-mediated cytolysis. The mechanisms underlying CD4^+ ^T-cell mediated killing during acute and persistent FV infection are not fully understood. Perforin, granzyme A, and granzyme B are effector molecules of the granule exocytosis pathway that are mainly produced by CD8^+ ^T cells and control acute FV infection. However, these molecules are not essential during the chronic phase while Fas-FasL interaction, a cytotoxic pathway that CD4^+ ^T cells can use [125,126], is mandatory for effective control of FV replication during persistent infection [127].

### CD4^+ ^regulatory T cells in FV infection

Pioneering work using the FV model established that mice persistently infected with FV display elevated levels of activated CD4^+^CD25^+ ^natural Tregs with potent inhibitory activity including the suppression of CD8^+ ^T cell-mediated killing of FV-induced tumors [60]. Later studies showed that FV-induced Tregs rapidly suppressed the function of TCR transgenic, FV-specific CD8^+ ^T cells adoptively transferred into chronically infected mice [61] (Figure 2). Kinetic studies indicated that deterioration in the ability of effector CD8^+ ^T cells to produce cytotoxic molecules and cytokines begins at 2 weeks post infection (wpi), the same time point when CD4^+ ^Treg expansion is peaking [62] (Figure 1). The kinetic properties of FV-mediated Treg expansion and CD8^+ ^T-cell dysfunction are not changed by LDV co-infection, and the expansion of Treg occurs during FV infection but not LDV infection [62,128]. To investigate the correlation between dysfunction of effector T cells and expansion of CD4^+ ^Tregs, the DEREG mouse was employed. These mice express a Diptheria Toxin (DT) receptor/GFP fusion gene under the control of the Foxp3 promoter, which is a transcription factor critical for the development and function of CD4^+ ^Tregs [129]. Foxp3 expressing cells can be experimentally depleted by treatment with DT. When FV infected DEREG mice receive DT, it leads to specific deletion of CD4^+^Foxp3^+ ^Tregs. During acute FV infection, Treg depletion results in strongly augmented peak CD8^+ ^T cell responses, including a rise in the frequency of FV-specific effector CD8^+ ^T cells, dramatically enhanced expression and degranulation of cytotoxic molecules, and increased *in vivo *CTL-mediated lysis of infected target cells [128]. Most importantly, this increase in CD8^+ ^T cell activity results in a significant reduction in virus loads. During chronic FV infection, ablation of Tregs induces proliferation of FV-specific CD8^+ ^T cells as well as reactivation of the residual but functionally exhausted CD8^+ ^T cells [39]. Importantly, the reactivation of the suppressed CD8^+ ^T cell response in Treg-depleted mice results in reduced viral set points during chronic retroviral infection.

CD4^+ ^natural Tregs also influence the outcome of CD4^+ ^effector T cell responses during acute FV infection. FV-specific CD4^+ ^T cells display anti-viral effector functions until 2 wpi, but thereafter their ability to produce IFNγ is reduced [67] (Figure 1). These FV-specific CD4^+ ^T cells with reduced IFNγ expression at 3 wpi regain their ability to produce IFNγ following depletion of CD4^+^Foxp3^+ ^Tregs in infected DEREG mice [67]. Thus, it is evident that CD4^+ ^Tregs negatively influence effector functions of CD4^+ ^T cells during acute FV infection thereby impairing initial control over viral replication [67,129]. These findings are supported by the work of Antunes *et al*., who showed that bone-marrow pathology observed in FV-infected lymphopenic mice, which is mediated by FV-specific CD4^+ ^T cells is inhibited by Tregs [32].

Expansion of CD4^+ ^Tregs during FV infection is highly compartmentalized with CD4^+ ^Tregs expanding in organs with high viral replication and associated inflammation. Interestingly, depletion experiments showed that the presence of CD8^+ ^T cells supports the expansion of CD4^+ ^Tregs in lymphatic tissues such as spleen and bone marrow [128]. Likewise, in HIV infection, CD4^+ ^Tregs expand predominantly in lymph nodes where the virus replicates most efficiently and virus-specific CD8^+ ^T cells accumulate. Thus, Treg numbers in lymph nodes correlate very well with disease progression of HIV infected individuals [36,55,130]. Moreover, increased expression of α_E_β_7 _integrin (CD103) on CD4^+^Foxp3^+ ^Tregs suggests a role of integrins in compartmentalization of Tregs in FV infection [62]. Hence, it becomes imperative to investigate local Treg responses before drawing conclusions on the role of Tregs in retroviral infections.

In addition to CD8^+ ^T cells, dendritic cells (DC) also seem to be involved in the expansion of Tregs. We have previously shown that FV-infected DCs do not fully mature and specifically expand Foxp3^+ ^Tregs *in vitro *[131]. This has also been described for HIV-infected DCs [132] suggesting a possible mechanism that retroviruses may use to increase numbers of Tregs at sites of infection. If DCs are involved in Treg expansion, one might presume that they present viral antigens to Tregs that then proliferate in an antigen-specific manner. However, FV-specific induced Tregs are undetectable in FV-infected mice either by using class II tetramers or after adoptive transfer of FV-specific CD4^+ ^T cells from TCR transgenic mice [128]. This finding is in agreement with the fact that CD4^+ ^T-cell mediated bone-marrow pathology observed in FV-infected lymphopenic hosts is impeded by immunosuppressive natural Tregs that are not specific for FV [32]. Recent findings from LCMV studies may explain these seemingly contradictory results. Tregs expanding after an LCMV clone 13 infection are not LCMV-specific, but at least a fraction of them expand in response to an endogenous retroviral superantigen (Sag) [133]. The chronic LCMV infection upregulates expression of the Sag in DCs, which then induce proliferation of Tregs with certain T cell receptors that can bind Sag. There is experimental evidence that the percentage of Tregs with the same T cell receptor (Vβ5) increases during FV infection (own unpublished results), so a similar mechanism may also be involved in the expansion of Tregs after FV infection.

Knowledge about the mechanisms underlying Treg-mediated immunosupression during FV infection is limited. Studies using transgenic mice have demonstrated that CD4^+^CD25^+ ^T cells isolated from mice chronically infected with FV suppress IFNγ and granzyme B production by activated CD8^+ ^T cells. Suppression occurs in a direct cell-to-cell contact dependent manner independent of the presence of APCs [134]. CD4^+ ^Tregs may do so via the expression of connexins, which are gap-junction proteins that have been found to be critical for the transfer of the potent inhibitory second messenger cyclic AMP (cAMP) into effector T cells [135,136]. In contrast, soluble factors such as IL-10 and transforming growth factor (TGF)-β secreted by CD4^+ ^Tregs do not contribute towards Treg-mediated immunosuppression in *in vitro *and *in vivo *experiments [61,134]. Furthermore, FV-induced Tregs do not secrete granzymes, ruling out granzyme-dependent Treg-mediated apoptosis of effector T cells [62]. The mechanism of suppression by Tregs in FV infected mice is still under investigation. In HIV infections, immunosuppressive IL-10 production by CD4^+ ^T cells has been associated with disease progression, but it is unclear whether these CD4^+ ^T cells were Tregs [137]. It has very recently been shown that Tregs control HIV replication in activated T cells via a contact-dependent mechanism involving cAMP [138].

Given the well-established role of Tregs in pathogen persistence, it is now of great interest to develop therapeutic approaches to manipulate this immunosuppressive subset of cells. Treg functions are reversed by blocking glucocorticoid-induced tumour necrosis factor receptor (GITR), a member of the TNF receptor superfamily. GITR is also a phenotypic marker of CD4^+^Foxp3^+ ^Tregs and it is highly expressed on Tregs during FV infection [62]. Blockade by antibodies leads to heightened production of IFNγ and TNFα by CD8^+ ^T cells [61]. Antibody-mediated signaling through CD137 (4-1BB), a co-stimulatory molecule also from the TNF receptor superfamily, renders CD8^+ ^T cells resistant to suppression by Tregs. Thus, anti-CD137 antibody therapy promotes virus-specific CD8^+ ^T cell proliferation and development of effector functions to exert control over chronic FV infections [139]. *In vitro *experiments with CD8^+ ^T cells from HIV-infected patients also show restored functional properties following treatment with anti-CD137 antibodies [140].

In mice, an alternative therapeutic approach is the depletion of Tregs, such as is done experimentally in the DEREG mouse experiments [39,58,128]. Depletion of Tregs leads to concerns that autoimmunity or other immunopathology might be induced, but transient depletion of Tregs in the DEREG mice is not associated with detectable immunopathology even during an ongoing antiretroviral immune response [128]. Such a therapeutic approach may be a possible treatment in HIV infected humans using an IL-2-toxin fusion protein (ONTAK) [141] that kills CD4^+^CD25^+ ^Tregs by binding to the IL-2 receptor via their expression of CD25. Treatment of cancer patients with ONTAK did not induce serious clinical side effects. Jiang and co-workers performed an interesting experiment to show that IL-2-toxin fusion protein-mediated depletion of CD4^+^CD25^+ ^Tregs in HIV-1 infected humanized mice resulted in a significant reduction of viral loads during acute HIV infection [142]. However, it is not known whether the reduction of viral loads is mediated by an enhanced immune response.

In addition to HIV, Treg-mediated dysfunction of effector T cells is a matter of concern in other chronic virus infections such as HCV, HBV, and EBV [143]. Therefore, therapeutic manipulation of Tregs *in vivo *with respect to enhancing virus-specific immunity and balancing immunopathology could have widespread clinical applications.

## Conclusion

It has been known for some time that CD4^+ ^T cells play a critical role in retroviral immunity, but only recently has the complexity of this subpopulation begun to be realized. Several distinct functions ascribed to subpopulations of CD4^+ ^T cell have now been defined in mouse retrovirus models. Type 1 helper CD4^+ ^T cells were important for the maintenance and survival of effector CD8^+ ^T cells, and follicular helper T cells critically supported antibody responses. CD4^+ ^T cells with direct antiviral activity were also described, mainly during chronic retroviral infection, but which may be active during acute infections as well. Concurrent with the kinetics of the antiviral CD4^+ ^T cell response during acute retroviral infection were the expansion and activation of a subpopulation of natural regulatory T cells at sites of infection. The natural regulatory T cells suppressed effector T cell responses, which interfered with immune control of virus replication and contributed to viral chronicity. Similar findings have also been made in HIV infected humans and the therapeutic manipulation of regulatory T cells *in vivo *with respect to enhancing retrovirus-specific immunity is a new frontier of high interest in the treatment of viral infections.

## Competing interests

The authors declare that they have no competing interests.

## Authors' contributions

SN, UD and KJH were responsible for drafting and revising the manuscript as well as organizing the content. WB, MJ-YP, and GK contributed significantly in drafting the manuscript and revising it critically. All authors read and approved the final manuscript.
